# Genome Sequence of the Microsporidian Species *Nematocida* sp1 Strain ERTm6 (ATCC PRA-372)

**DOI:** 10.1128/genomeA.00905-14

**Published:** 2014-09-18

**Authors:** Malina A. Bakowski, Margaret Priest, Sarah Young, Christina A. Cuomo, Emily R. Troemel

**Affiliations:** a Division of Biological Sciences, Section of Cell and Developmental Biology, University of California, San Diego, La Jolla, California, USA; b Broad Institute of MIT and Harvard, Cambridge, Massachusetts, USA

## Abstract

Microsporidia comprise a phylum of obligate intracellular pathogens related to fungi. Microsporidia *Nematocida* sp1 strain ERTm6 was isolated from wild-caught *Caenorhabditis briggsae* and causes a lethal intestinal infection in *Caenorhabditis* nematodes. We report the genome sequence of *N.* sp1 ERTm6, which will facilitate study of the *Nematocida* genus and other Microsporidia.

## GENOME ANNOUNCEMENT

Microsporidia are parasites that are ubiquitous in the environment and infect virtually all animal phyla, including humans. Phylogenomic analysis revealed that microsporidia are in the earliest branching group of sequenced fungi and that *Nematocida* is in the earliest branching group of sequenced microsporidia (1–3). Outside of their hosts, microsporidia persist as environmentally resistant spores. They infect by deploying an infection apparatus (polar tube), through which spore contents are delivered into host cells. Microsporidia replicate intracellularly as meronts, which differentiate into spores and are shed from the host into the environment. The discovery of *Nematocida parisii*, a microsporidian species that infects the model organism *Caenorhabditis elegans* in the wild (4), opened up new avenues for investigating microsporidia pathogenesis (2, 5). Several *N. parisii* and *N.* sp1 strains have been isolated from wild-caught *Caenorhabditis* nematodes throughout the world (6). Sequence analysis of two *N. parisii* (ERTm1 and ERTm3) and one *N.* sp1 genome (ERTm2) provided the first molecular evidence that microsporidia are likely to be diploid, with extensive heterozygote regions (2). *N.* sp1 was particularly polymorphic in these regions (1 single nucleotide polymorphism [SNP] every 82 bp). Thus, we sought to investigate the genome of a second *N.* sp1 strain for comparison.

*N.* sp1 ERTm6 was found infecting wild-caught *Caenorhabditis briggsae* JU1638, collected from soil next to a yam/taro plant in Cape Verde Islands (17.13768 N, 25.06689 W). It was subsequently isolated and transferred into the *C. elegans* wild-type N2 strain for propagation and harvest (4). Total DNA was extracted from *Nematocida*-infected *C. elegans* using standard nematode lysis, followed by phenol/chloroform extraction methods (2). The purity and concentration of DNA was checked using NanoDrop ND-8000 UV-Vis (Thermo Fisher Scientific). For genome sequencing, we constructed two genome shotgun libraries with average insert sizes of 178 bases and 2.6 kb and sequenced both using Illumina technology (7, 8). Reads were assembled with ALLPATHS-LG version R41828 (9), generating a consensus sequence with a read depth of roughly 118×.

The genome size was estimated to be 4.28 Mb, with a GC content of 38.30%. The assembly was organized in 57 contigs, linked by paired-end reads into 24 scaffolds. As found for other sequenced *Nematocida* (2), this is a compact genome and 67.48% of the genome sequence is predicted to be coding, with a mean distance between coding sequences of 578.63 bp. The average base is found in a scaffold with an *N*_50_ of 797.7 kb and a contig with an *N*_50_ of 219.3 kb. A total of 2,433 protein-coding genes, 51 tRNA genes, and 9 rRNA genes were predicted as previously described (2).

We found that *N.* sp1 ERTm6 is likely diploid, although the heterozygous regions are not as polymorphic as ERTm2. The ERTm6 strain has 1 SNP every 989 bp, identified from BWA-MEM (10) alignments of the Illumina reads using the GATK Unified Genotyper version 2.7 (11). This genome, together with the ERTm1, ERTm2, and ERTm3 genomes, provide an excellent resource for investigating microsporidia diversity and coevolution of parasites and hosts.

### Nucleotide sequence accession numbers.

This whole-genome shotgun project has been deposited at DDBJ/EMBL/GenBank under the accession number AKIJ00000000. The version described in this paper is version AKIJ01000000.

## References

[1] Capella-Gutiérrez S Marcet-Houben M Gabaldón T 2012 Phylogenomics supports microsporidia as the earliest diverging clade of sequenced fungi. BMC Biol. 10:47. 10.1186/1741-7007-10-47 22651672 PMC3586952

[2] Cuomo CA Desjardins CA Bakowski MA Goldberg J Ma AT Becnel JJ Didier ES Fan L Heiman DI Levin JZ Young S Zeng Q Troemel ER 2012 Microsporidian genome analysis reveals evolutionary strategies for obligate intracellular growth. Genome Res. 22:2478–2488. 10.1101/gr.142802.112 22813931 PMC3514677

[3] James TY Pelin A Bonen L Ahrendt S Sain D Corradi N Stajich JE 2013 Shared signatures of parasitism and phylogenomics unite cryptomycota and microsporidia. Curr. Biol. CB 23:1548–1553. 10.1016/j.cub.2013.06.057 23932404

[4] Troemel ER Félix MA Whiteman NK Barrière A Ausubel FM 2008 Microsporidia are natural intracellular parasites of the nematode *Caenorhabditis elegans*. PLoS Biol. 6:2736–2752. 10.1371/journal.pbio.0060309 19071962 PMC2596862

[5] Bakowski MA Desjardins CA Smelkinson MG Dunbar TL Lopez-Moyado IF Rifkin SA Cuomo CA Troemel ER 2014 Ubiquitin-mediated response to microsporidia and virus infection in *C. elegans*. PLoS Pathog. 10:e1004200 24945527 10.1371/journal.ppat.1004200 PMC4063957

[6] Bakowski MA Luallen RJ Troemel ER 2014 Chapter 13 Microsporidia infections in *Caenorhabditis elegans* and other nematodes. *In* Weiss LM Becnel JJ (ed), Microsporidia: pathogens of opportunity. John Wiley & Sons, Chichester, UK. 10.1002/9781118395264.ch13

[7] Fisher S Barry A Abreu J Minie B Nolan J Delorey TM Young G Fennell TJ Allen A Ambrogio L Berlin AM Blumenstiel B Cibulskis K Friedrich D Johnson R Juhn F Reilly B Shammas R Stalker J Sykes SM Thompson J Walsh J Zimmer A Zwirko Z Gabriel S Nicol R Nusbaum C 2011 A scalable, fully automated process for construction of sequence-ready human exome targeted capture libraries. Genome Biol. 12:R1. 10.1186/gb-2011-12-1-r1 21205303 PMC3091298

[8] Grad YH Lipsitch M Feldgarden M Arachchi HM Cerqueira GC Fitzgerald M Godfrey P Haas BJ Murphy CI Russ C Sykes S Walker BJ Wortman JR Young S Zeng Q Abouelleil A Bochicchio J Chauvin S Desmet T Gujja S McCowan C Montmayeur A Steelman S Frimodt-Møller J Petersen AM Struve C Krogfelt KA Bingen E Weill FX Lander ES Nusbaum C Birren BW Hung DT Hanage WP 2012 Genomic epidemiology of the *Escherichia coli* O104:H4 outbreaks in Europe, 2011. Proc. Natl. Acad. Sci. U. S. A. 109:3065–3070. 10.1073/pnas.1121491109 22315421 PMC3286951

[9] Gnerre S Maccallum I Przybylski D Ribeiro FJ Burton JN Walker BJ Sharpe T Hall G Shea TP Sykes S Berlin AM Aird D Costello M Daza R Williams L Nicol R Gnirke A Nusbaum C Lander ES Jaffe DB 2011 High-quality draft assemblies of mammalian genomes from massively parallel sequence data. Proc. Natl. Acad. Sci. U. S. A. 108:1513–1518. 10.1073/pnas.1017351108 21187386 PMC3029755

[10] Li H Durbin R 2009 Fast and accurate short read alignment with Burrows–Wheeler transform. Bioinformatics 25:1754–1760. 10.1093/bioinformatics/btp324 19451168 PMC2705234

[11] McKenna A Hanna M Banks E Sivachenko A Cibulskis K Kernytsky A Garimella K Altshuler D Gabriel S Daly M DePristo MA 2010 The Genome Analysis Toolkit: a MapReduce framework for analyzing next-generation DNA sequencing data. Genome Res. 20:1297–1303. 10.1101/gr.107524.110 20644199 PMC2928508

